# Pre-Transplant Frequencies of FoxP3^+^CD25^+^ in CD3^+^CD8^+^ T Cells as Potential Predictors for CMV in CMV-Intermediate Risk Kidney Transplant Recipients

**DOI:** 10.3389/ti.2024.12963

**Published:** 2024-05-29

**Authors:** Agnes A. Mooslechner, Max Schuller, Verena Pfeifer, Konstantin A. Klötzer, Barbara Prietl, Alexander H. Kirsch, Philipp Stiegler, Robert Sucher, Harald Sourij, Alexander R. Rosenkranz, Kathrin Eller

**Affiliations:** ^1^ Division of Nephrology, Department of Internal Medicine, Medical University of Graz, Graz, Austria; ^2^ Otto Loewi Research Center, Division of Pharmacology, Medical University of Graz, Graz, Austria; ^3^ Center for Biomarker Research in Medicine, CBmed GmbH, Graz, Austria; ^4^ Division of Endocrinology and Diabetology, Department of Internal Medicine, Medical University of Graz, Graz, Austria; ^5^ Division of General, Visceral and Transplant Surgery, Department of Surgery, Medical University of Graz, Graz, Austria

**Keywords:** kidney transplant, biomarker, cytomegalovirus management, immune cells, valganciclovir

## Abstract

Cytomegalovirus (CMV) infection detrimentally influences graft survival in kidney transplant recipients, with the risk primarily determined by recipient and donor serostatus. However, recipient CD8^+^ T cells play a crucial role in CMV control. The optimal preventive strategy (prophylaxis vs. pre-emptive treatment), particularly for seropositive (intermediate risk) recipients, remains uncertain. We investigated CD8^+^ T cell subpopulation dynamics and CMV occurrence (DNAemia ≥ 100 IU/mL) in 65 kidney transplant recipients, collecting peripheral blood mononuclear cells before (T1) and 1 year after transplantation (T2). Comparing the two timepoints, we found an increase in granulocyte, monocyte and CD3^+^CD8^+^ T cells numbers, while FoxP3^+^CD25^+^, LAG-3^+^ and PD-1^+^ frequencies were reduced at T2. CMV DNAemia occurred in 33 recipients (55.8%) during the first year. Intermediate risk patients were disproportionally affected by posttransplant CMV (*N* = 29/45, 64.4%). Intermediate risk recipients developing CMV after transplantation exhibited lower leukocyte, monocyte, and granulocyte counts and higher FoxP3^+^CD25^+^ frequencies in CD3^+^CD8^+^ T cells pre-transplantation compared to patients staying CMV negative. Pre-transplant FoxP3^+^CD25^+^ in CD3^+^CD8^+^ T cells had the best discriminatory potential for CMV infection prediction within the first year after transplantation (AUC: 0.746). The FoxP3^+^CD25^+^ CD3^+^CD8^+^ T cell subset may aid in selecting intermediate risk kidney transplant recipients for CMV prophylaxis.

## Graphical Abstract

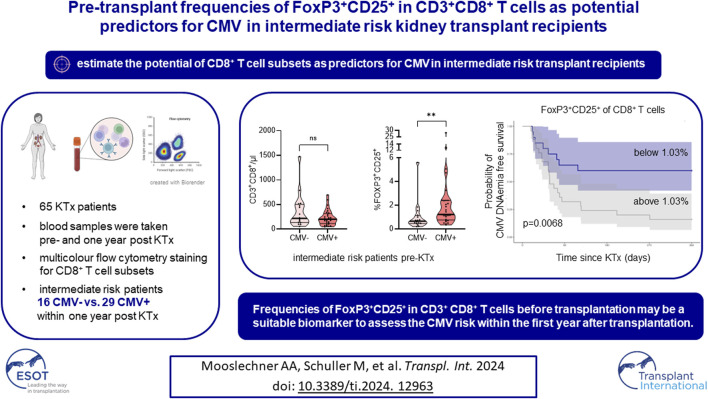

## Introduction

Cytomegalovirus (CMV) infection is a common complication in kidney transplant recipients (KTR). The disease spectrum encompasses asymptomatic replication to potentially life-threatening CMV disease, defined as end-organ affection or flu-like symptoms accompanied by fever and hematological abnormalities [1]. Furthermore, indirect effects of CMV include increased rates of transplant rejection, graft loss, and death [2–5].

Following often asymptomatic primary infection, CMV is not eliminated but remains a latent infection in non-hematopoietic cells. CD8^+^ T cells are crucial for the control of primary infection and reactivation. Antiviral mechanisms of these cells include the production of cytokines and cytotoxic granules directed at infected cells [6]. However, subpopulations of CD8^+^ T cells, including FoxP3^+^ cells, are associated with suppressing cytotoxicity, potentially counteracting an efficient antiviral response [7]. CMV drives the terminal differentiation and expansion of CD8^+^ T cells, leading to long-term changes in the composition of the CD8^+^ T cell compartment [8].

CMV DNAemia may arise in immunosuppressed individuals [9], and the risk of posttransplant CMV is primarily dependent on the serostatus of the recipient (R) and the donor (D). While either R+/D+ or R+/D− are considered intermediate risk, R−/D+ are at high risk of CMV infection. R−/D− are at the lowest risk of posttransplant CMV [10, 11]. Other risk factors highlight the role of an intact immune system for CMV control and include T-cell depleting immunosuppression, high-dose mycophenolate mofetil (MMF) or mycophenolic acid (MPA), high-dose corticosteroids and lymphocytopenia [10, 11].

A preventive strategy is recommended for those who are at intermediate and high risk of CMV [1, 12]. Both antiviral chemoprophylaxis and preemptive treatment are viable options with different advantages and problems [1, 12]. Whereas prophylaxis is often complicated by post-prophylaxis CMV disease, the treatment threshold for CMV DNAemia is unknown [13]. The preemptive treatment approach requires frequent screening (preferably weekly) and poses a substantial logistic burden [14], while prophylaxis is more costly [15]. Moreover, valganciclovir prophylaxis harbors the risk of myelotoxicity [16, 17], potentially rendering transplant recipients vulnerable to breakthrough CMV and other infections.

Additionally, valganciclovir dosing is dependent on kidney function, and insufficient dosing can lead to breakthrough CMV [18]. Although prophylaxis and preemptive treatment have shown similar efficacy in preventing CMV disease and indirect CMV effects like rejection [19], many centers, including ours, employ a prophylactic strategy for high-risk recipients (R-/D+). Of note, an extended chemoprophylaxis for 200 days compared to 100 days in high-risk patients did not only lead to a reduction of CMV disease, but was also associated with fewer rejections and opportunistic infections [20]. For intermediate risk constellation, considerable uncertainty regarding the optimal preventive strategy exists. Improved identification of vulnerable individuals would allow for personalized prophylaxis beyond CMV D and R serostatus.

In this study, we followed the dynamics of the peripheral immune cell composition of a cohort of kidney transplant recipients before transplantation and 1 year thereafter. We set a strong focus on the CD8^+^ T cell compartment and its subpopulations associated with immunoregulatory functions (FoxP3), ageing (CD28), and exhaustion (LAG-3, PD-1). Intrigued by the interplay of CMV and CD8^+^ T cells, we hypothesized that the pre-transplant peripheral CD8^+^ T cell pool may harbor prognostic subsets for the susceptibility to CMV DNAemia post-transplantation.

## Materials and Methods

### Study Design and Study Population

We screened 105 CKD G5 patients prior to transplantation, as previously described [21]. Briefly, adult (age ≥ 18 years) kidney transplant recipients without prevalent immunosuppression who received an organ from a deceased donor after obtaining written informed consent were included. Blood samples were drawn before the dialysis session before transplant surgery (T1) and 1 year after transplantation (T2). Only those with complete follow-up and intact graft at T2 were included in the final analysis.

The study protocol was approved by the Institutional Review Board of the Medical University of Graz, Austria (28- 514ex15/16). The study was registered as #DRKS00026238 in the German Register of Clinical Studies.

### CMV Prophylaxis, Screening, and Treatment

According to local standards, individuals at high risk of CMV, including R+/D-constellation, following anti-thymocyte globulin (ATG) induction or ATG rejection treatment, were selected for 3 months of CMV prophylaxis with valganciclovir. The valganciclovir dose adjustment was performed as recommended in the package insert for impaired kidney function.

CMV positive (CMV+) individuals were defined as CMV PCR ≥ 100 international units (IU)/mL in EDTA-plasma at least once during the first posttransplant year measured by cobas^®^ 5800 (Roche Holding, Basel, Switzerland) at the Diagnostic and Research Institute of Hygiene, Microbiology, and Environmental Medicine at the Medical University Graz. CMV PCR testing in peripheral blood was performed at every regular outpatient visit during the first year. Routine visit frequencies according to the local center standard are month 1—weekly, month 2–3—every 2–3 weeks, month 4–6—monthly, month 6–12—every 4–6 weeks. If clinically indicated, patients were checked more frequently including CMV PCR testing.

Those without any CMV PCR ≥ 100 IU/mL in the first year were defined as CMV negative (CMV−).

In case of CMV DNAemia with ≥ 100 IU/mL, potential strategies encompassed observation of viral replication, dose reduction or temporary discontinuation of MMF/MPA and/or the initiation of therapeutic dose valganciclovir. The selected strategy was subject to the discretion of the treating physician and was contingent upon the specific clinical circumstances. Antiviral treatment was administered until two consecutive PCR results showed < 100 IU/mL.

Duration of CMV positivity describes the interval between the first PCR result ≥ 100 IU/mL and the last PCR result ≥ 100 IU/mL. Any subsequent PCR with ≥ 100 IU/mL after the initial episode was defined as relapse. If there was a solitary PCR with ≥ 100 IU/mL, the duration of CMV positivity was designated as 1 day. The definition of CMV disease adhered to current recommendations [1]. The highest PCR measurement in IU/mL during the first year was designated as CMV peak.

### PBMC Isolation and Flow Cytometry

Peripheral blood mononuclear cells (PBMC) were isolated at T1 and T2 as described previously [21]. Briefly, fresh heparinized whole blood samples were collected in BD vacutainer tubes containing lithium heparin (Becton Dickinson, Franklin Lakes, NJ, United States) and diluted at 1:1 ratio with phosphate-buffered saline (PBS), and then carefully layered into a tube preloaded with Lymphoprep density gradient media (Stemcell Technologies, Vancouver, Canada). Following a density gradient centrifugation process (20 min, 800 × g at room temperature), the PBMC layer was collected and subsequently washed with PBS. Viability and cell count were determined using an automated dual fluorescence cell counter (LUNA-FL, Logos Biosystems, Anyang, South Korea) prior to multi-parameter staining of 1 × 10^6^ cells per fluorescence-activated cell sorting (FACS) panel. Additionally, 0.5 × 10^6^ cells were designated for an unstained control. Furthermore, 50 μL of fresh whole blood was subjected to staining with anti-CD45 APC-H7 antibodies (Becton Dickinson), with the addition of 123 count eBeads (Thermo Fisher Scientific, Waltham, MA, United States) for analyzing absolute numbers of leukocyte subpopulations. Absolute cell numbers were calculated according to manufacturer’s instructions. Absolute numbers of subpopulations were determined by multiplying the absolute counts of lymphocytes by the respective frequencies of each subpopulation relative to total lymphocytes.

BD Lyse/Fix buffer (Becton Dickinson) was used for surface panel staining according to the manufacturer’s protocol. All antibodies were obtained from BD and details are summarized in Supplementary Table S1. Sample acquisition occurred on a four-laser BD FACS Fortessa SORP instrument (Becton Dickinson). Data analysis was performed using FlowJo software Version 10.10.0 (Becton Dickinson). Compensation utilized UltraComp eBeads (Thermo Fisher Scientific), and fluorescence minus one (FMO) controls were implemented. Our gating strategy is illustrated in Supplementary Figures S1, S2.

### Statistical Analysis

Statistical analysis and graphical representations were done using Statistical Package for Social Sciences (SPSS v27, SPSS Inc., Chicago, IL, United States), GraphPad Prism 8.0.1 (GraphPad Software Inc., San Diego, CA, United States), and R Studio (Version 4.2.2, PBC, Boston, MA, United States). Normality was assessed by Kolmogorov-Smirnov test. Flow cytometry data are shown in violin plots and median and interquartile range (IQR) are indicated. For categorical data, absolute values and relative frequencies (%) are given. Differences between two independent groups were calculated with t-tests, Mann-Whitney U-tests, and χ^2^-tests, as appropriate. Paired groups were compared using dependent t-test or Wilcoxon signed-rank test for normal and non-normal variables.

Receiver operating characteristics (ROC) curves and area under the receiver operating characteristic curves (area under the curve, AUC) were derived using *pROC* (v. 1.18.4) for R studio [22]. Youden indices were determined for each predictor variable, aiming to identify optimal cutoff points that maximize sensitivity and specificity. Positive predictive values (PPV) and negative predictive values (NPV) were calculated to assess the performance of each variable. DeLong method was used to calculate 95% confidence intervals for AUCs. Probability of CMV DNAemia was calculated with *survminer* (v. 0.4.9). Differences in CMV DNAemia free survival probability were assessed using log-rank test [23].

*p*-values below 0.05 were defined as significant without adjustment for multiple testing.

## Results

### Study Population

**FIGURE 1:**
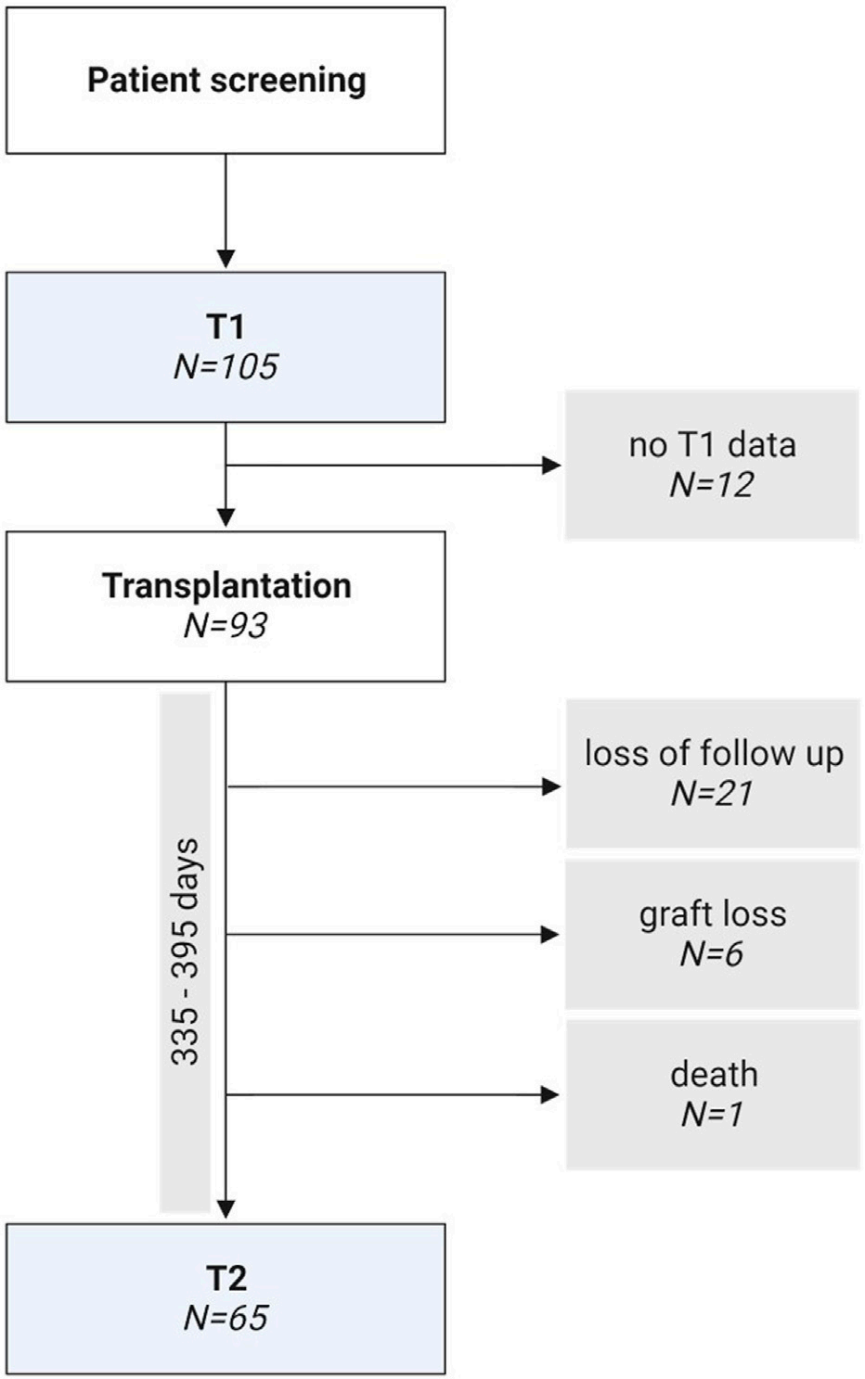
Flow chart of the study design.

One hundred and five prospective kidney transplant recipients met our inclusion criteria. Due to loss of follow-up, unavailable flow cytometric data, graft loss, and death, 40 patients had to be excluded, and 65 patients remained for the final analysis (Figure 1). Loss of follow-up was primarily due to patients leaving our center, thus not adhering to local standard-of-care including frequency of visits as well as flow cytometry evaluation 1 year after transplantation. Reasons for graft loss included severe transplant rejection (*N* = 4), polyoma nephropathy (*N* = 1) and surgical complications (*N* = 1).

Demographic and baseline clinical data are summarized in Table 1. Induction therapy consisted of anti-thymocyte globulin (ATG) or basiliximab (BX), depending on the immunological risk [21]. This was followed by standard triple immunosuppression with tacrolimus (TAC), mycophenolate mofetil (MMF) or mycophenolic acid (MPA) and corticosteroids, apart from one individual who received cyclosporin A (CyA) instead of TAC, and another recipient, who did not tolerate MMF/MPA and was switched to azathioprine (AZA).

**TABLE 1:** Descriptive statistics of KTRs with 1-year follow up.

*N*	65
Age (years)	56 (47–63.5)
Caucasian Ethnicity	59 (90.8)
Male gender	40 (61.5)
BMI (kg/m^2^)	26.1 (21.8–29.3)
Preemptive	4 (6.2)
HD/PD	50/11 (76.9/16.9)
Diabetes mellitus	12 (18.5)
Donor Age (years)	55 (47.5–70.5)
ECD	35 (53.8)
HLA Mismatches	
0	2 (3.1)
1	4 (6.2)
2	4 (6.2)
3	15 (23.1)
4	30 (46.2)
5	10 (15.4)
6	0
Kidney disease	
Diabetes	11 (16.9)
Hypertensive	3 (4.6)
Glomerular	16 (24.6)
Cystic	11 (16.9)
Other	24 (36.9)
Immunosuppression	
ATG	7 (10.8)
BX	58 (89.2)
CS	65 (100)
CyA	1 (1.5)
Tac	64 (98.5)
MMF/MPA	64 (98.5)
AZA	1 (1.5)

Data are presented as median and IQR or absolute values and percentages, depending on the variable; BMI, body mass index; HD, hemodialysis; PD, peritoneal dialysis; ECD, extended criteria donor; ATG, anti-thymocyte globulin; BX, basiliximab; CS, corticosteroids; CyA, cyclosporin A; Tac, tacrolimus; MMF, mycophenolate mofetil; MPA, mycophenolic acid; AZA, azathioprine.

For CMV serostatus, 9 high-risk (R-/D+, 13.8%), 11 low-risk (R-/D-, 16.9%), and 45 intermediate risk (R+, 69.2%) patients were included.

### Dynamics of Immune Cell Populations and CD8 Subpopulations in Pre- and 1-Year Post-Transplant Recipients

**FIGURE 2:**
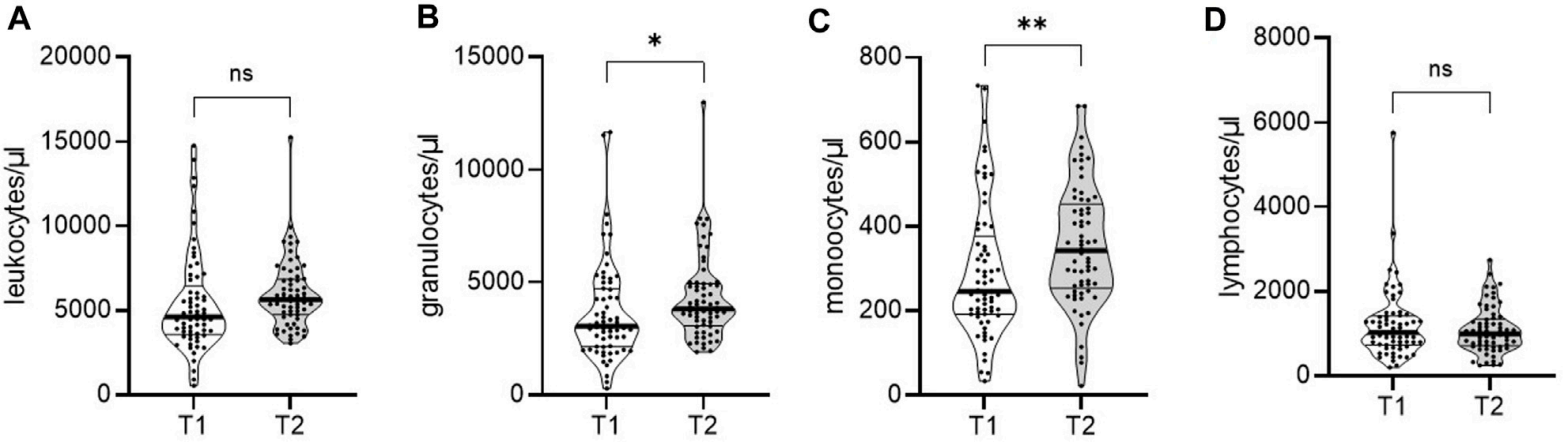
Dynamics of immune cell populations in a cohort of end-stage kidney disease patients to 1-year post-transplant. Whole blood of 65 patients was analyzed by flow cytometry pre- (T1) and 1-year post-transplantation (T2). Violin plots show the data distribution of absolute numbers of **(A)** leukocytes, **(B)** granulocytes, **(C)** monocytes, and **(D)** lymphocytes. Each black dot represents data of one patient. All data are represented in median (heavy black line) and IQR (thin black line). Statistical analysis was calculated with Wilcoxon signed-rank test (*p < 0.05; **p < 0.01).

To study the effect of kidney transplantation on immune cell populations, we analyzed a cohort of CKD G5 patients before and 1-year after transplantation. Absolute leukocyte numbers were unchanged 1 year after transplantation (Figure 2A). However, absolute numbers of granulocytes and monocytes were significantly increased compared to pre-transplant (Figures 2B, C). Overall lymphocyte numbers were comparable at both time points (Figure 2D).

**FIGURE 3:**
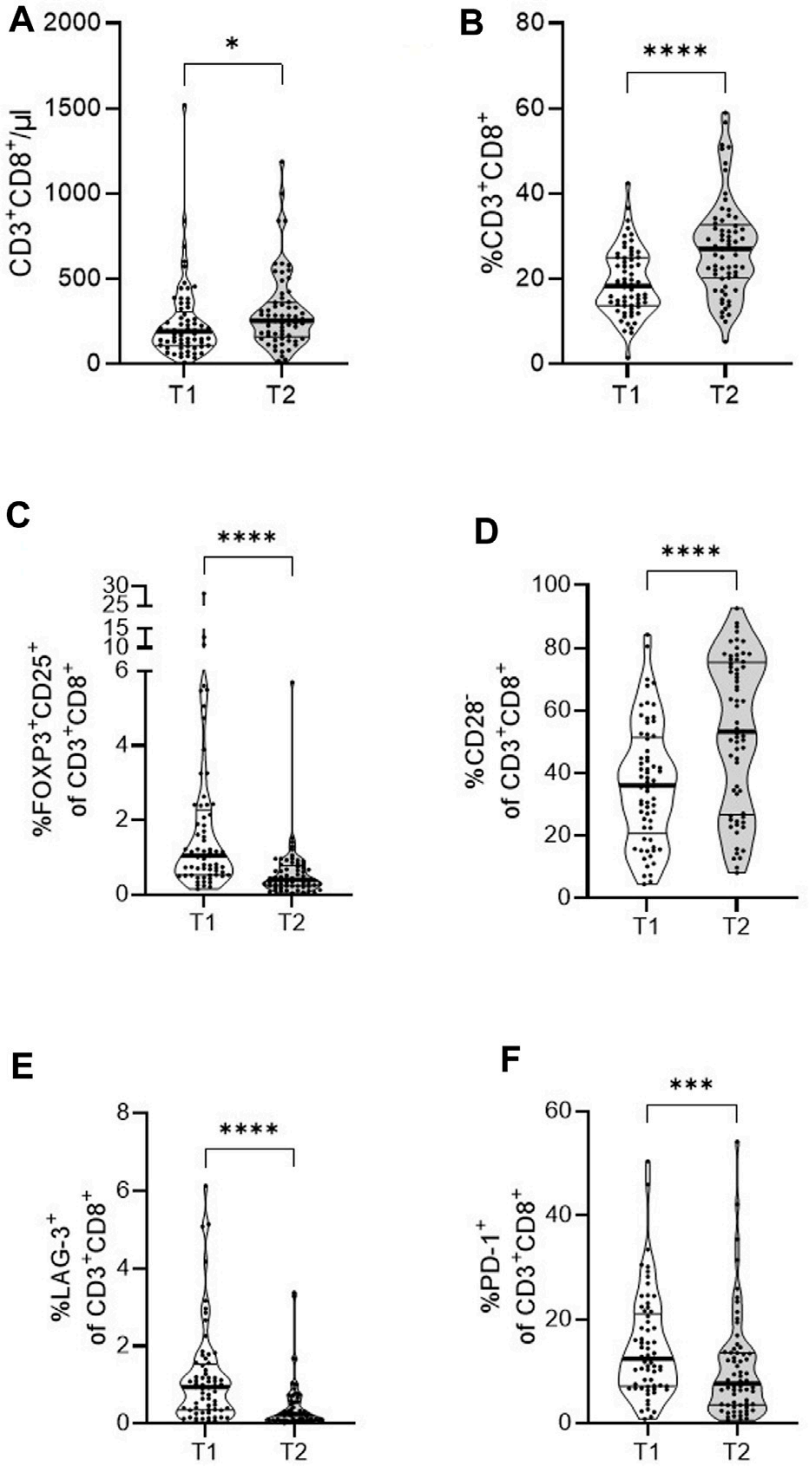
Dynamics of CD8^+^ T cells and subsets in a cohort of end-stage kidney disease patients to 1-year post-transplant. PBMCs of 65 patients were analyzed by flow cytometry pre- (T1) and 1-year post-transplantation (T2). Violin plots show the data distribution of absolute numbers of **(A)** CD8^+^ T cells and frequencies of **(B)** CD8^+^ T cells, **(C)** FoxP3^+^CD25^+^, **(D)** CD28^−^, **(E)** LAG-3^+^, and **(F)** PD-1^+^ CD8^+^ T cells. Each black dot represents data of one patient. All data are represented in median (heavy black line) and IQR (thin black line). Statistical analysis was calculated with Wilcoxon signed-rank test (*p < 0.05; ***p < 0.001; ****p < 0.0001).

Next, we investigated the absolute CD8^+^ T cell numbers and relative percentages of CD8^+^ T cell subpopulations in kidney transplant recipients before and 1 year after transplantation. Overall, absolute numbers and percentages of CD8^+^ T cells were increased 1 year after kidney transplantation (Figures 3A, B). Additionally, the composition in the CD8^+^ T cell population changed after transplantation. Frequencies of FoxP3^+^CD25^+^ were decreased (Figure 3C), while frequencies of CD28^−^ cells were increased (Figure 3D). Both the LAG-3^+^ and PD-1^+^ subpopulations were significantly decreased compared to pre-transplantation (Figures 3E, F).

### Graft Function Is Reduced in Kidney Transplant Recipients After CMV Infection

We retrospectively investigated the incidence of CMV within our study cohort and found CMV replication defined as CMV PCR ≥ 100 IU/mL in 33 patients (50.7%) during the first year. Juxtaposition of CMV− and CMV+ individuals showed no difference in age, BMI, underlying kidney disease, or immunosuppressive treatment (Table 2). We observed a non-significant trend for women and non-Caucasians to be more frequently affected by CMV. Most strikingly, intermediate risk patients were particularly affected by CMV (87.9% intermediate risk patients in overall CMV+), while high-risk patients and prophylaxis were evenly distributed between both groups. Of note, rejection episodes were seen at similarly low frequencies in the CMV− and CMV+ groups (Table 2). Furthermore, donor age and extended criteria donors (ECD), as defined by Port et al. [24], were comparable between groups (Table 2).

**TABLE 2:** Comparison of KTRs who remained CMV− and those who become CMV+ within the first year of transplantation.

*N*	CMV−	CMV+	*p*-value
	32	33	
Age (years)	57.5 (49.5–63.75)	53 (46–63.5)	0.295
Caucasian Ethnicity	31 (96.9)	28 (84.8)	0.094
Male gender	23 (71.9)	17 (51.5)	0.092
BMI (kg/m^2^)	26.4 (22.6–31.2)	25.5 (21.6–27.6)	0.200
Preemptive	2 (6.3)	2 (6.1)	0.975
HD/PD	22/8 (68.8/25)	28/3 (84.8/9.1)	0.124/0.087
Diabetes mellitus	7 (21.9)	5 (15.2)	0.485
Donor Age (years)	54.5 (44.3–69.8)	59 (49–71)	0.478
ECD	16 (50)	19 (57.6)	0.540
BK-Polyoma viremia	8 (25)	5 (15.2)	0.321
CMV constellation			<0.001
R−/D−	11 (34.4)	0	
R+/D−	9 (28.1)	4 (12.1)	
R+/D+	7 (21.9)	23 (69.7)	
R+/D?	0	2 (6.1)	
R−/D+	5 (15.6)	4 (12.1)	
CMV prophylaxis	7 (21.9)	9 (27.3)	0.614
Kidney disease			
Diabetes	7 (21.9)	4 (12.1)	0.294
Hypertensive	1 (3.1)	2 (6.1)	0.573
Glomerular	7 (21.9)	9 (27.3)	0.614
Cystic	6 (18.8)	5 (15.2)	0.699
Other	11 (34.4)	13 (39.4)	0.675
Immunosuppression			
ATG	3 (9.4)	4 (12.1)	0.721
BX	29 (90.6)	29 (87.9)	0.721
CS	32 (100)	33 (100)	1.000
CyA	0	1 (3)	0.321
Tac	32 (100)	32 (97)	0.321
MMF/MPA	31 (96.9)	33 (100)	0.306
AZA	1 (3.1)	0	0.306
Rejection within first year	6 (18.8)	6 (18.2)	0.953
Kidney function after 1 year			
Serum-urea (mg/dL)	49.5 (37.5–55)	54 (45–79.5)	0.009
Serum-creatinine (mg/dL)	1.25 (1.08–1.57)	1.66 (1.39–2.06)	<0.001
eGFR (ml/min/1.73m^2^)	56.5 (45.8–70.7)	43.4 (28.9–50.7)	<0.001

Continuous variables are depicted as mean and IQR, categorical variable as absolute and relative frequencies; BMI, body mass index; HD, hemodialysis; PD, peritoneal dialysis; ECD, extended criteria donor; ATG, anti-thymocyte globulin; BX, basiliximab; CS, corticosteroids; CyA, cyclosporin A; Tac, tacrolimus; MMF, mycophenolate mofetil; MPA, mycophenolic acid; AZA, azathioprin; eGFR, estimated glomerular filtration rate.

Upon closer investigation of CMV positivity, we found that the median time to positivity was 57 days (28–82 days) after transplantation, and the median duration of CMV DNAemia was 6 days (1–23 days). Median CMV peak was 1500 IU/mL (490–4,950 IU/mL). Only 11 patients were symptomatic, while the majority remained asymptomatic. CMV treatment consisted of valganciclovir in 24 cases (72.7%). Concomitantly with antiviral treatment, antimetabolite dose was reduced in 18 patients (54.5%), or antimetabolite treatment was paused in three patients (9.1%) (Table 3). Nine patients (27.3%) were managed without antiviral treatment. CMV DNAemia in these patients was asymptomatic and was cleared either spontaneously (*N* = 4, 12.1%) or by reduction of immunosuppression alone (*N* = 5, 15.2%). CMV positivity in the setting of valganciclovir prophylaxis was observed in nine patients (27.3%). Four of those patients were at high-risk for CMV (12.1%). The other five KTRs received valganciclovir prophylaxis following treatment for acute rejection (15.2%). In four of these patients (12.1%) CMV developed as a breakthrough infection within 90 days of treatment, and one patient became CMV positive 194 days after diagnosis of rejection (Table 3).

**TABLE 3:** Characteristics of CMV infection in all 33 CMV+ KTRs.

*N*	33
Time to positivity (d)	57 (28–81.5)
CMV high risk constellation (%)	4 (12.1)
Valganciclovir prophylaxis (%)	9 (27.3)
Valganciclovir prophylaxis dose (mg/d)	225 (225–450)
Rejection prior CMV (%)	5 (15.2)
ATG rejection treatment ≤90 days prior CMV (%)	4 (12.1)
CMV peak (IU/mL)	1,500 (490–4,950)
Symptomatic CMV disease (%)	11 (33.3)
Duration of positivity (d)	6 (1–23)
Recipients with relapses (%)	10 (30.3)
Watch and wait (%)	4 (12.1)
MMF/MPA dose reduction without antiviral therapy (%)	4 (12.1)
MMF/MPA pause without antiviral therapy (%)	1 (3)
Valganciclovir therapy without MMF/MPA dose reduction/pause (%)	3 (9.1)
Valganciclovir therapy with MMF/MPA dose reduction (%)	18 (54.5)
Valganciclovir therapy with MMF/MPA pause (%)	3 (9.1)
Valganciclovir dose (mg/d)	450 (281–450)
Letermovir therapy (%)	1 (3)
Letermovir dose (mg/d)	480

Median and IQR are given for continuous and absolute values and relative frequencies for categorical variables, respectively. ATG, anti-thymocyte globulin; MMF, mycophenolate mofetil; MPA, mycophenolic acid.

Despite the low threshold for the definition of CMV infection, kidney function was reduced in CMV+ after 1 year as evidenced by serum urea, serum creatinine, and creatinine-based estimated glomerular filtration rate (eGFR) (Table 2).

### Circulating Leukocyte Numbers as Potential Predictors of CMV in Intermediate Risk Individuals Before Kidney Transplantation

**FIGURE 4:**
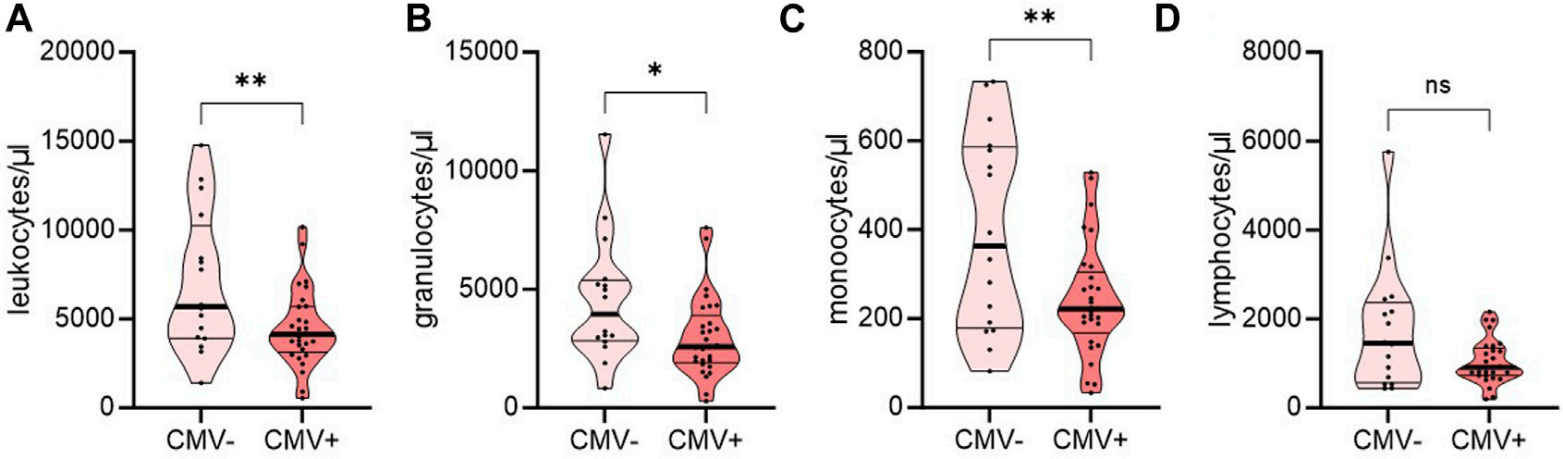
Distribution of immune cell populations in a cohort of end-stage kidney disease patients with intermediate-risk for CMV DNAemia pre-transplant. Intermediate-risk patients were grouped regarding their affection by CMV DNAemia post-transplantation. Whole blood of 16 CMV− and 29 CMV+ patients was analyzed by flow cytometry pre-transplant (T1). Violin plots show the data distribution of absolute numbers of **(A)** leukocytes, **(B)** granulocytes, **(C)** monocytes, and **(D)** lymphocytes. Each black dot represents data of one patient. All data are represented in median (heavy black line) and IQR (thin black line). Statistical analysis was calculated with Student’s t-Test or Mann-Whitney-U Test after testing for normal distribution (*p < 0.05; **p < 0.01).

Recognizing the pronounced risk of R+ for CMV and the reduced graft function after CMV (Supplementary Table S2), we aimed to estimate the potential of circulating leukocyte numbers as predictors of CMV infection in these patients (16 CMV− and 29 CMV+), specifically. We compared the abundance of leukocyte subpopulations before transplantation of intermediate risk individuals not affected and affected by CMV DNAemia post-transplantation. Individuals who tested CMV+ during the first year of transplantation had significantly lower numbers of overall leukocytes, granulocytes, and monocytes (Figures 4A–C). The abundance of overall lymphocytes was comparable pre-transplant regardless of later CMV DNAemia (Figure 4D).

### Pre-Transplant FoxP3^+^CD25^+^ in CD3^+^CD8^+^ T Cells as Potential Predictors of CMV in Intermediate Risk Transplant Recipients

**FIGURE 5:**
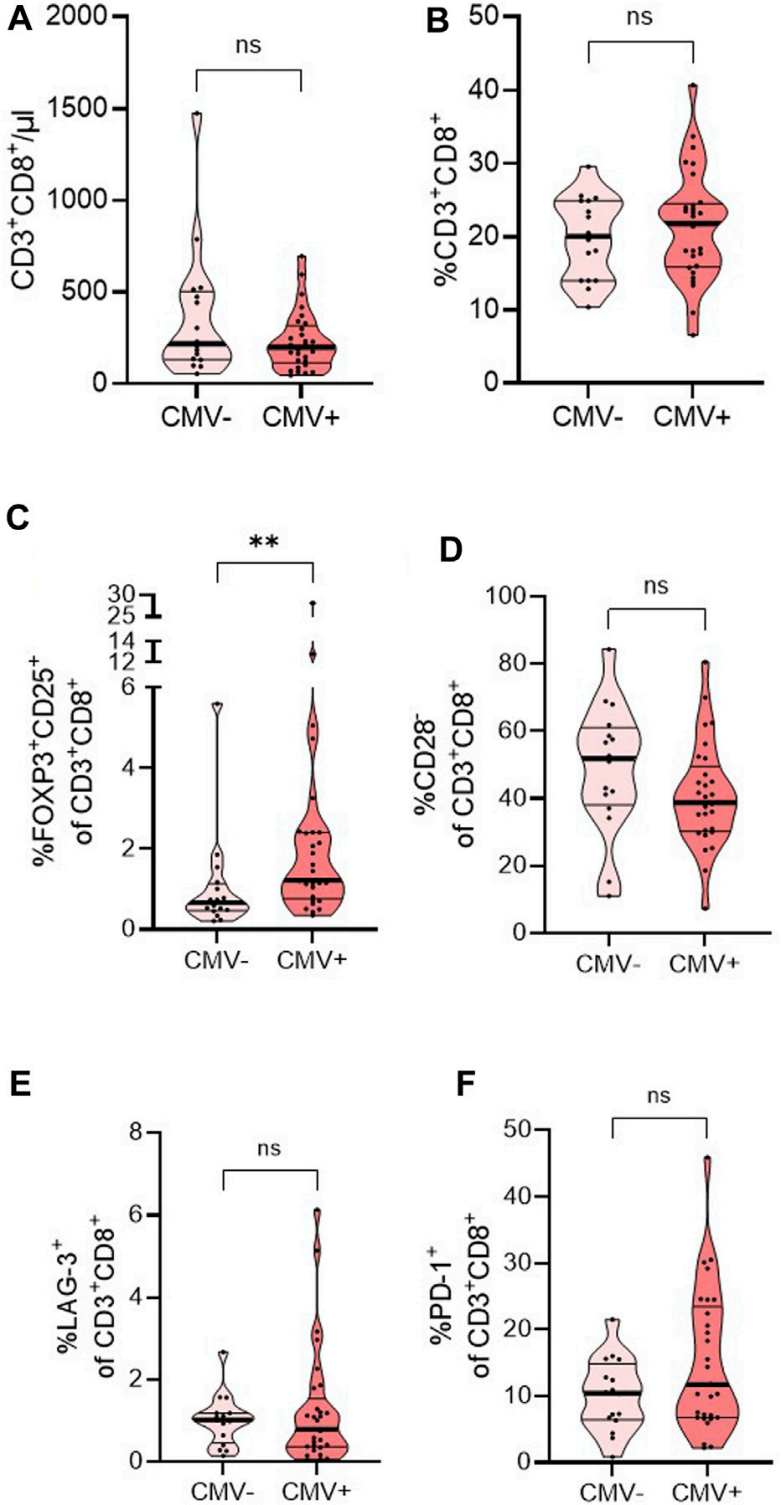
Distribution of CD8^+^ T cells and subsets in a cohort of end-stage kidney disease patients with intermediate risk for CMV DNAemia pre-transplant. Intermediate-risk patients were grouped regarding their affection by CMV DNAemia post-transplantation. Whole blood of 16 CMV− and 29 CMV+ patients was analyzed by flow cytometry pre-transplant (T1). Violin plots show the data distribution of absolute numbers of **(A)** CD8^+^ T cells and frequencies of **(B)** CD8^+^ T cells, **(C)** FoxP3^+^CD25^+^, **(D)** CD28^−^, **(E)** LAG-3^+^, and **(F)** PD-1^+^ CD8^+^ T cells. Each black dot represents data of one patient. All data are represented in median (heavy black line) and IQR (thin black line). Statistical analysis was calculated Mann-Whitney-U Test after testing for normal distribution (**p < 0.01).

Before transplantation, intermediate risk individuals had comparable numbers and frequencies of CD8^+^ T cells (Figures 5A, B). In individuals with intermediate risk who tested positive for CMV within 12 months post-transplantation, the percentage of FoxP3^+^CD25^+^ was significantly increased before transplantation (Figure 5C), while frequencies of CD28^−^, LAG-3^+^, and PD-1^+^ among CD3^+^CD8^+^ T cells were not different pre-transplantation (Figures 5D–F) compared to those who remained negative for CMV.

**FIGURE 6:**
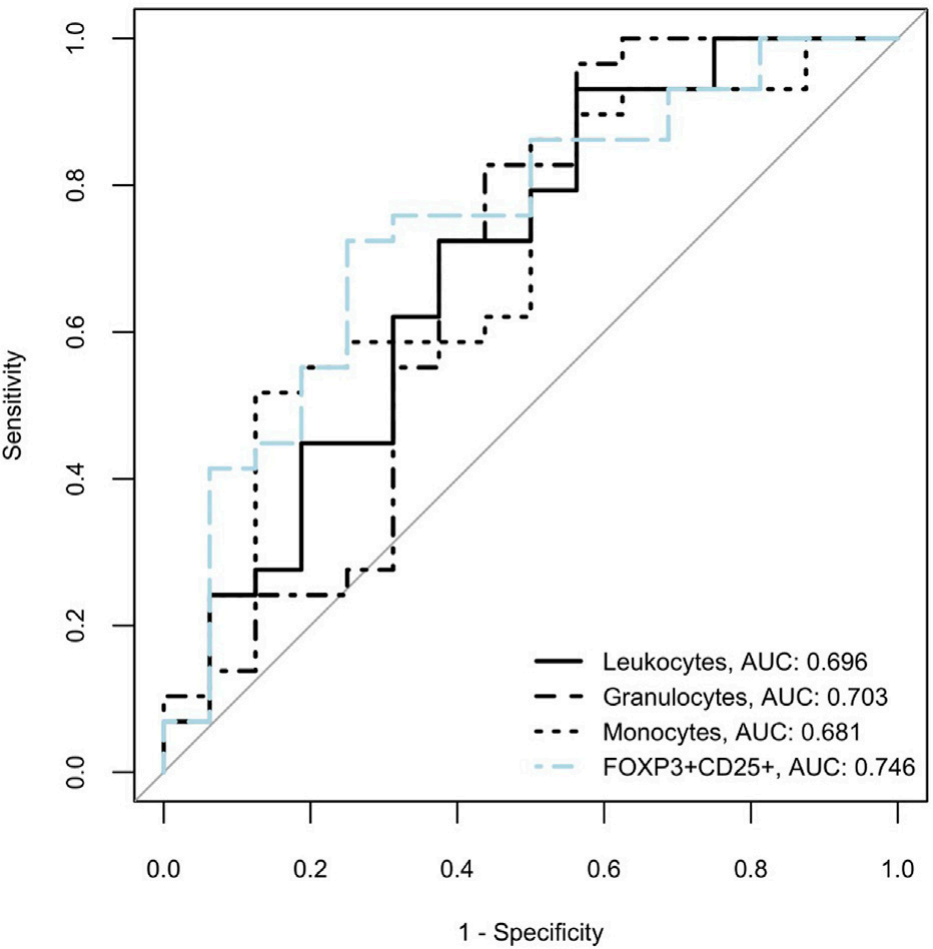
Receiver operating characteristic (ROC) curves for cellular subsets for development of CMV infection in intermediate risk KTRs are shown. Areas under the receiver operating characteristic curves (area under the curve, AUC) are listed next to the variable names in the figure legend.

Next, we compared potential pre-transplant predictors, namely, leukocytes, granulocytes, monocytes, and FoxP3^+^CD25^+^CD3^+^CD8^+^ T cells. Utilizing Youden indices, we aimed to estimate sensitivity and specificity. Our findings revealed comparable areas under the curve (AUC) for all subsets. While Youden-derived thresholds for leucocytes and monocytes offered great sensitivity, 93% and 96%, respectively, specificities with either marker were low (43.8% and 43.8%, respectively). The cutoff for granulocytes, on the contrary, showed good specificity (87.5%) but poor sensitivity (51.7%) for CMV DNAemia. A cutoff of 1.03% for FoxP3^+^ CD25^+^ in CD3^+^ CD8^+^ T cells, allowed for the best discrimination between those who would become CMV positive and those who would remain CMV negative with a balanced sensitivity and specificity of 72.4% and 75%, respectively (Table 4; Figure 6).

**TABLE 4:** Comparison of leucocytes and specific subsets with predictive potential for CMV infection.

Potential predictor	AUC (95% CI)	Youden index	Youden’s threshold	Sensitivity	Specificity	PPV	NPV
Leukocytes	0.696 (0.524–0.868)	0.368	7448.744	0.931	0.438	0.75	0.778
Granulocytes	0.703 (0.537–0.868)	0.392	2578.271	0.517	0.875	0.882	0.5
Monocytes	0.681 (0.494–0.868)	0.403	519.655	0.966	0.438	0.757	0.875
FoxP3^+^CD25^+^ (% of CD3^+^CD8^+^)	0.746 (0.591–0.901	0.474	1.03	0.724	0.75	0.864	0.6

AUC, area under the curve; CI, confidence interval; PPV, positive predictive value; NPV, negative predictive value.

**FIGURE 7:**
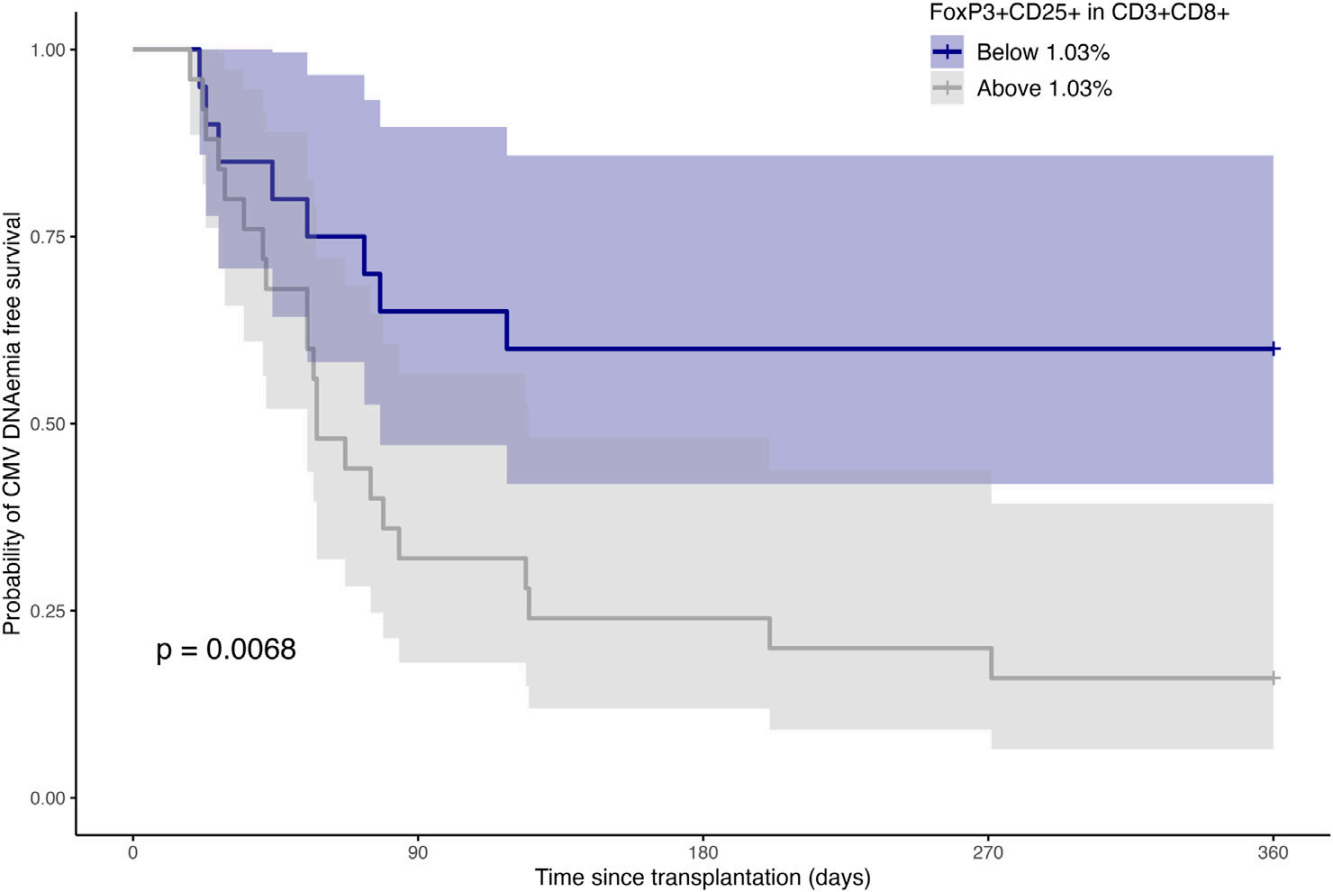
CMV DNAemia-free survival probability over time post-transplantation, stratified by levels of FoxP3^+^CD25^+^ within CD3^+^CD8^+^ T cells. Shaded areas constitute the 95% confidence intervals. Log-rank test shows a significant difference in probability of CMV DNAemia between groups (“Below 1.03%” vs. “Above 1.03%”).

Further exploration involved the stratification of intermediate risk patients based on the frequency of FoxP3^+^CD25^+^ in CD3^+^CD8^+^ T cells, employing the established 1.03% cutoff. Results indicated a consistent distribution across demographic factors (age, donor age, BMI), with notably higher prevalence of CMV infection in the group with higher frequencies (Supplementary Table S3). While not achieving statistical significance, variations in the use of ATG as induction treatment were noted between the two groups. Probability of CMV DNAemia was higher in KTRs with pretransplant percentages of FoxP3^+^CD25^+^ in CD3^+^CD8^+^ T cells above 1.03 (Figure 7).

### Consequences of CMV DNAemia on Leukocyte and CD8^+^ T Cell Populations 1-Year Post-Kidney Transplantation

One-year post-transplantation, individuals showed no significant differences in leukocyte, granulocyte, and monocyte numbers regardless of CMV infection status (Supplementary Figures S3A–S3C). However, individuals who tested positive for CMV post-transplant had a higher number of lymphocytes 1 year after transplantation (Supplementary Figure S3D).

CMV DNAemia had lasting effects on the CD8^+^ T cell compartment 1 year after transplantation. CMV+ individuals showed increased CD8^+^ T cell numbers and frequencies of CD3^+^CD8^+^ T cells (Supplementary Figures S4A, B). While frequencies of FoxP3^+^CD25^+^ cells were comparable to CMV- individuals (Supplementary Figure S4C), frequencies of CD28^−^ cells among CD3^+^CD8^+^ T cells were significantly increased 1-year post-transplant (Supplementary Figure S4D). Additionally, in CMV+ individuals, the frequencies of LAG-3^+^ among CD3^+^CD8^+^ T cells were elevated (Supplementary Figure S4E), while the percentage of PD-1^+^ was not changed (Supplementary Figure S4F).

## Discussion

We studied the dynamics of immune cell composition, focusing on CD8^+^ T cells before and 1-year after transplantation. The study was based on immune cell phenotyping by flow cytometry to analyze overall leukocyte, granulocyte, monocyte, and CD8^+^ T cell numbers, as well as frequencies of CD8^+^ T cell subpopulations associated with immunoregulatory functions (FoxP3), ageing (CD28), and exhaustion (LAG-3, PD-1). Our goal was to find a prognostic phenotypic pattern of post-transplant CMV DNAemia that might be considered to help identify intermediate risk individuals who would benefit from CMV prophylaxis.

Deteriorating kidney function in CKD is associated with decreased T cell numbers, which can be attributed mainly to a reduction of naïve T cells and an increase of CD8^+^ memory T cells [25]. In line with these results, we show higher overall CD8^+^ T cell numbers and frequencies 1 year after transplantation compared to pre-transplantation. However, the composition within the CD8^+^ T cell population is changed. While we did not see an increase in frequencies of FoxP3^+^CD25^+^ regulatory CD8^+^ T cells, we saw a significant increase of CD28^−^ CD8^+^ T cells 1 year after transplantation. This finding confirms previous studies showing that these cells were significantly increased in dialysis patients compared to healthy controls and expanded further after kidney transplantation [26, 27], presumably due to continued antigen exposure [8]. CD28 is a co-stimulatory receptor on T cells mediating activation, proliferation, and longevity. The expression of CD28 declines with age, and these cells are discussed to have impaired effector functions [28].

Inhibitory receptors are negative regulators of immunopathology by counteracting T cell activation and peripheral tolerance [29]. Higher and sustained expression of LAG-3 and PD-1 are associated with T cell exhaustion, which weakens responses to infections. We show that 1-year after transplantation frequencies of LAG-3 and PD-1 in CD8^+^ T cells are significantly decreased in our cohort. These findings add to previous studies reporting around 1% of exhausted CD8^+^ T cells after 3 months post-transplantation, with an increase after CMV infection [27]. Moran et al., however, report increased PD-1 expression on CD8^+^ T cells 1 year after transplantation in a pediatric cohort, which was the opposite in our adult cohort [30]. Among others, loss of co-stimulatory receptors and upregulation of inhibitory receptors associated with exhaustion are hallmarks of T cell ageing [31]. Premature immune cell ageing has previously been reported in patients with CKD G5 [25]. Our findings suggest a partly reversed CD8^+^ T cell ageing phenotype due to reduced exhaustion markers after kidney transplantation. On the other hand, an increase in CD28^−^CD8^+^ T cells could indicate accelerated CD8^+^ T cell ageing in transplanted individuals compared to CKD G5.

Apart from the direct effects, CMV infection after transplantation is associated with reduced graft and patient survival [32]. CMV DNAemia was common in our cohort when a cutoff of ≥ 100 IU/mL was applied as the threshold. A major problem in CMV surveillance is the lack of a definitive threshold for significant CMV DNAemia [1, 12]. This issue is aggravated by the low comparability of CMV PCR testing platforms between centers [33]. Thus, determining a CMV PCR threshold for intervention underlies the physician’s judgement and depends on the clinical context. Our cutoff aligns with practice at our institution for therapeutic considerations. This comparably low threshold may explain our cohort’s low numbers of symptomatic infections and CMV end-organ disease [34]. Nonetheless, kidney function was reduced in those who had experienced CMV positivity by our standard, while other predictors of graft outcome (i.e., donor age and proportion of ECD) were similar between CMV+ and CMV−.

We focused on intermediate risk recipients as they were particularly affected by post-transplant CMV. Additionally, considerable uncertainty regarding the risk-to-benefit ratio of CMV prophylaxis in this subgroup exists. Particularly leukopenia is a common and severe complication with valganciclovir [35–37], and less myelotoxic alternatives have not been tested yet in intermediate risk KTRs [38].

R+ patients affected by CMV DNAemia after transplantation showed lower overall numbers of leukocytes, granulocytes, and monocytes pre-transplantation. Furthermore, these patients displayed higher frequencies of FoxP3^+^CD25^+^ CD8^+^ T cells pre-transplantation. FoxP3^+^CD25^+^CD3^+^CD8^+^ T cells are proposed to be a regulatory subpopulation within the CD8^+^ T cell compartment and have been shown to be able to suppress effector CD8^+^ and CD4^+^ T cell functions in part by IL-10 production, as well as induce inhibitory receptors on DCs potentially dampening immune responses against infections [39].

Previous studies have investigated the predictive potential of CMV-specific CD8^+^ T cells for various CMV-related endpoints [40–48]. Addressing the issue of intermediate risk recipients specifically, the absence of a pre-transplant CD8^+^ T cell response to the immediate early (IE)-1 antigen has been shown to predict post-transplant CMV [47, 48]. Furthermore, an increased abundance of CMV-specific IFNγ^+^ CD8^+^ T cells reduced the risk of high-level DNAemia and the necessity of treatment in a cohort of R+ solid organ transplant recipients [45].

Although these studies have shown promising results, their generalizability may be limited by small and heterogenous cohorts of different organ transplants and immunosuppressive regimens.

While our cohort is also small, it uniformly consists of kidney transplant recipients with similar immunosuppression. Moreover, monitoring FoxP3^+^CD25^+^CD3^+^CD8^+^ T cells is appealing, given its ease of implementation without the requirement for assessing CMV-specificity or conducting functional assays.

Interestingly, in our cohort, the increase in CD8^+^ T cell numbers and frequencies, as well as CD28 loss at year one after transplantation, are driven by CMV DNAemia. Patients testing continuously negative during the course of 1 year after transplantation show comparable numbers of these CD8^+^ T cell populations to pre-transplantation (data not shown), as also reported by Wang et al. [26].

Beyond the single-center nature and small sample size, several other limitations need to be addressed. We only included patients who underwent their posttransplant follow-up at our center ensuring relatively standardized frequency of visits and PCR testing. We focused on patients with a complete follow-up to allow for an equal time at risk for CMV in all patients. However, generalizability of our results may be compromised by inclusion and exclusion criteria. Similarly, the lack of an external validation cohort is a major limitation, and our findings need to be confirmed in an independent analysis. We specifically focused on CMV intermediate risk kidney transplant recipients, recognizing their susceptibility to frequent CMV infections and the uncertainties surrounding CMV prevention. Due to the already modest number of intermediate risk patients, we refrained from controlling for CMV prophylaxis, prior rejection, and ATG treatment in this subcohort. Although these factors were rare, we acknowledge that these may influence CMV reactivation. One limitation is the fact that, that we did not correct for multiple testing, because of the rather low n-number of flow cytometry analysis performed in this small cohort.

In summary, we found a substantially altered CD8^+^ T cell pool in kidney transplant recipients compared to the CKD G5 setting prior to transplantation. CD28^−^CD8^+^ T cells were expanded especially in patients after CMV DNAemia, while expression of regulatory and exhaustion markers was reduced after 1-year post-transplant. Determination of frequencies of FoxP3^+^CD25^+^ in CD3^+^CD8^+^ T cells already before transplantation may be a suitable biomarker to assess the CMV risk within the first year after transplantation and might thereby assist in the selection of intermediate risk individuals for CMV prophylaxis. Our findings need to be confirmed in an independent validation cohort. The outlook of CMV prophylaxis approach based on FoxP3^+^CD25^+^ assessment warrants consideration for investigation in a prospective trial.

## Data Availability

The raw data supporting the conclusion of this article will be made available by the authors, without undue reservation.
