# Supplementary material for: Pre-Transplant Frequencies of FoxP3+CD25+ in CD3+CD8+ T Cells as Potential Predictors for CMV in CMV-Intermediate Risk Kidney Transplant Recipients
Source: Transpl Int. 2024 May 29;37:12963. doi: 10.3389/ti.2024.12963 (PMC11167633; doi:10.3389/ti.2024.12963)
Supplement: Supplementary file 1 [file DataSheet1.PDF]

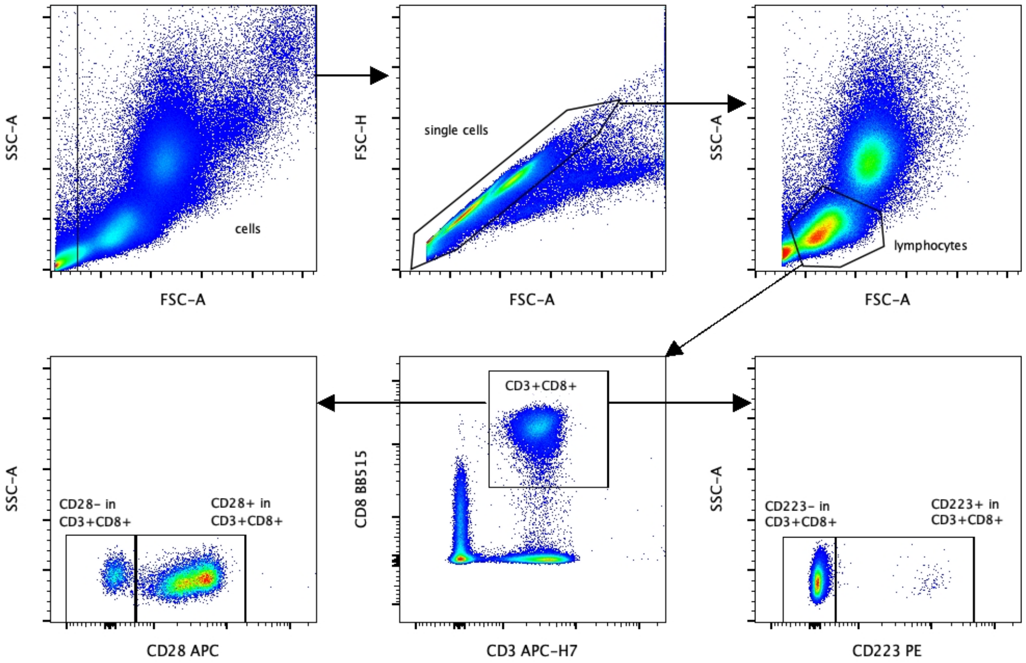

Supplementary Fig. 1. Representative dot plots for the gating strategy of Panel 1 identifying CD8 T cells and CD28^{-} and CD223^{+} (LAG-3) subsets. Lymphocytes were gated according to morphology (SSC-A vs. FSC-A), and doublets were excluded (via FSC-H and FSC-A). CD8^{+} T cells were identified as CD3 and CD8 double-positive cells. This population was further gated into CD28 positive and negative cells and CD223 positive and negative cells.

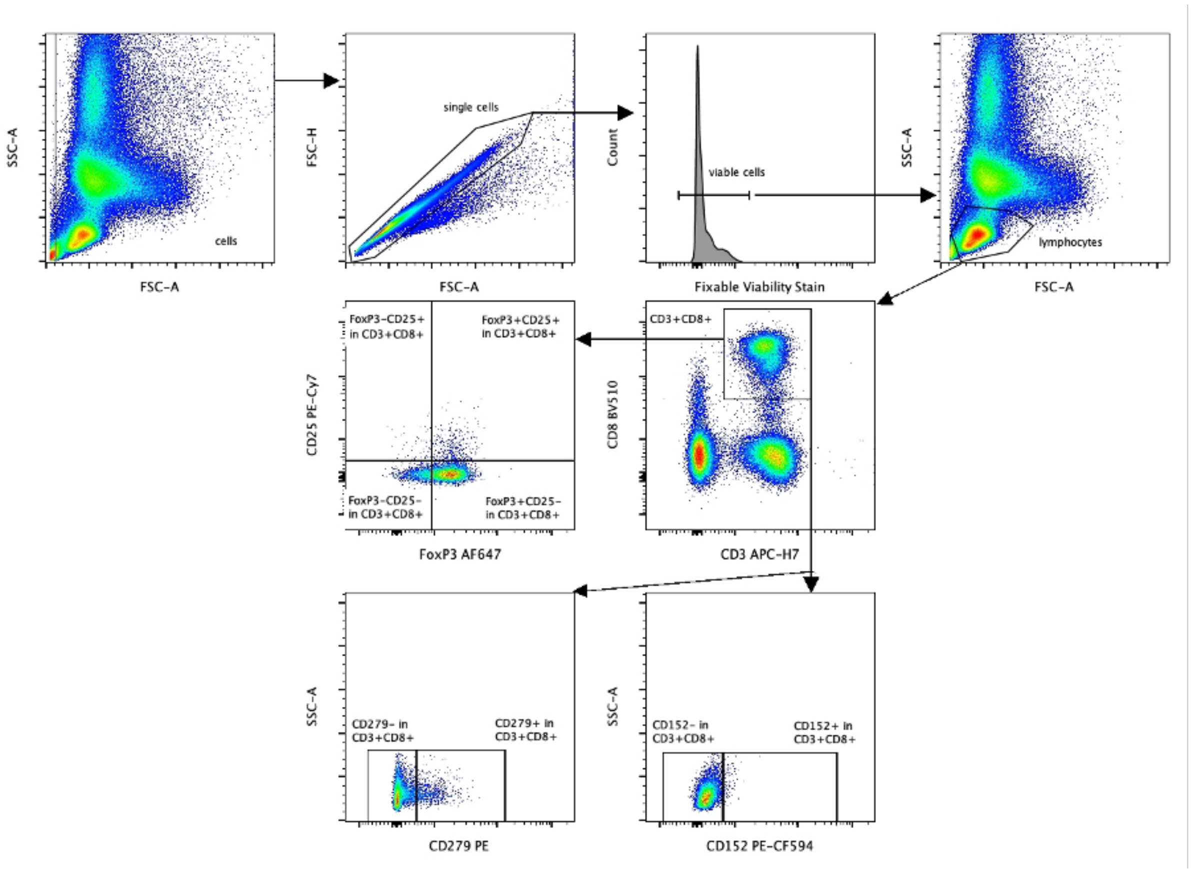

Supplementary Fig. 2. Representative dot plots for the gating strategy of Panel 2 identifying CD8 T cells and FoxP3^{+}CD25+ and CD279+ (PD-1) subsets. Lymphocytes were gated according to morphology (SSC-A vs. FSC-A), doublets were excluded (via FSC-H and FSC-A), and a fixable viability stain was used to identify living cells. CD8^{+} T cells were identified as CD3 and CD8 double-positive cells. This population was further gated according to their CD25 and FoxP3 expression. Additionally, CD3^{+}CD8^{+} T cells were gated for CD279 expression.

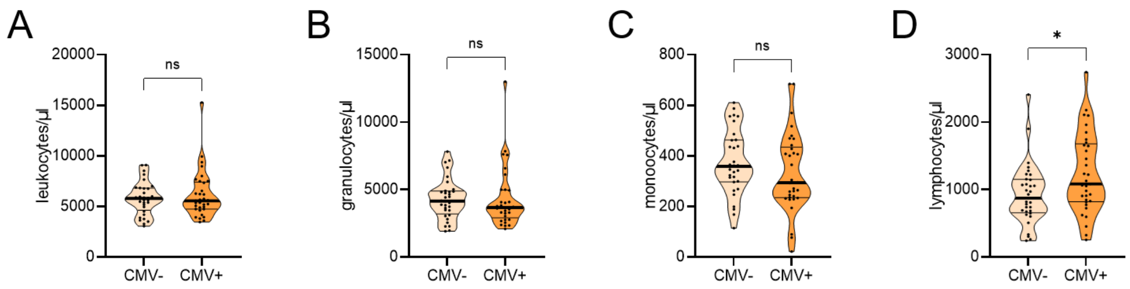

Supplementary Fig.3: Distribution of immune cell populations in a cohort of KTRs grouped according to CMV DNAemia during the one-year post-transplant. Whole blood of 32 CMV- and 33 CMV+ patients was analyzed by flow cytometry one-year post-transplant (T2). Violin plots show the data distribution of absolute numbers of (A) leukocytes, (B) granulocytes, (C) monocytes, and (D) lymphocytes. Each black dot represents data of one patient. All data are represented in mean (heavy black line) and IQR (thin black line). Statistical analysis was calculated with Mann-Whitney-U Test after testing for normal distribution (*p<0.05).

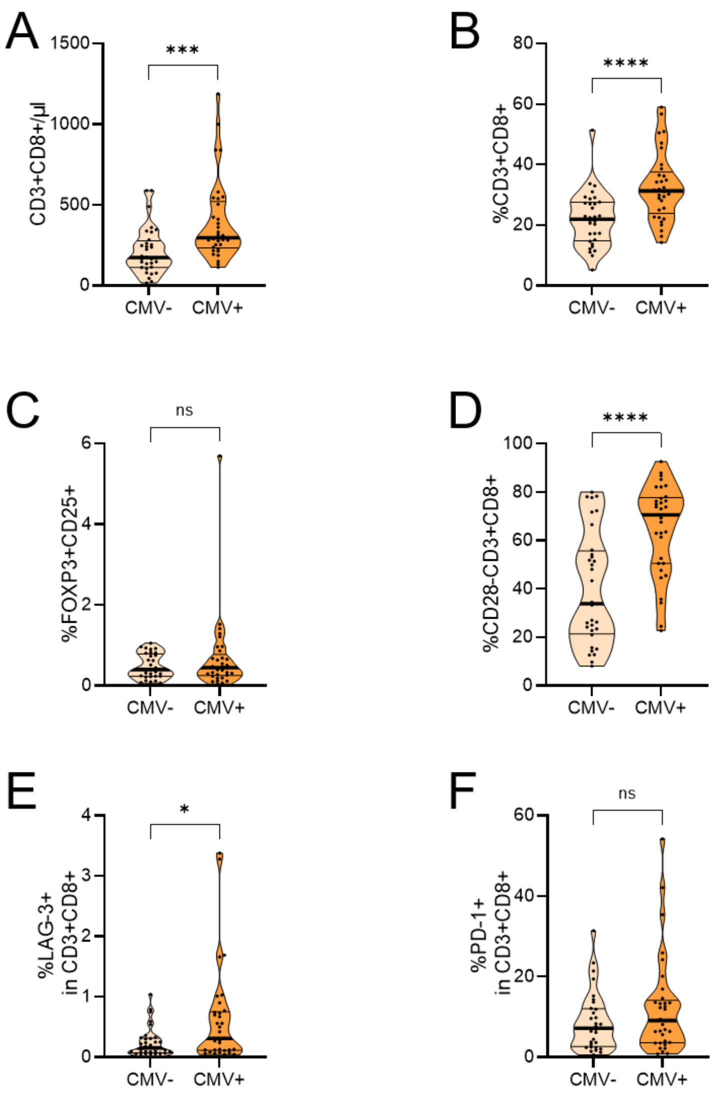

Supplementary Fig. 4: Distribution of CD8⁺ T cells and subsets in a cohort of KTRs grouped according to CMV DNAemia during the one-year period post-transplant. Whole blood of 32 CMV- and 33 CMV+ patients was analyzed by flow cytometry one-year post-transplant (T2). Violin plots show the data distribution of absolute numbers of (A) $CD8^+$ T cells and frequencies of (B) $CD8^+$ T cells, (C) $FoxP3^+CD25^+$, (D) $CD28^-$, (E) LAG-3^+, and (F) PD-1^+ $CD8^+$ T cells. Each black dot represents data of one patient. All data are represented in mean (heavy black line) and IQR (thin black line). Statistical analysis was calculated Student's *t*-Test or Mann-Whitney-U Test after testing for normal distribution (*p<0.05; ***p<0.001; ****p<0.0001).

	Clone	Fluorophor	Catalog
**Panel 1**			
CD8	RPA- T8	BB515	564526
CD223 (LAG3)	T47-530	PE	565616
CD28	28.2	APC	559770
CD3	SK7	APC-H7	560176
**Panel 2**			
CD8	SK1	BV510	563919
CD279 (PD-1)	EH12.1	PE	560795
CD152	BNI 3	PE-CF594	562742
CD25	2A3	PE-Cy7	335824
FoxP3	259D/C7	AF647	560045
FVS		APC-R700	564997
CD3	SK7	APC-H7	560176

Supplementary Table 1. Antibodies and clones used for CD8 T cell staining for Panel 1 and Panel 2.

	CMV -	CMV +	p-Value
N	*16*	*29*	
Age (years)	61 (51.3 – 66.3)	54 (47 – 64.5)	0.209
Caucasian Ethnicity	15 (93.8)	24 (82.8)	0.299
Male gender	11 (68.8)	14 (43.8)	0.186
BMI (kg/m^2)	27 (21.2 – 30.6)	25.2 (21.3 – 27.3)	0.200
Preemptive	1 (6.3)	2 (6.9)	0.934
HD/PD	12 (75)/3 (18.8)	24 (82.8)/3 (10.3)	0.533/0.427
Diabetes mellitus	3 (18.8)	3 (10.3)	0.427
Donor Age (years)	54.5 (34.3 – 69.8)	56 (49 – 70)	0.661
ECD	8 (50)	17 (58.6)	0.577
BK-Polyoma viremia	4 (25)	4 (13.8)	0.347
CMV prophylaxis	1 (6.3)	5 (17.2)	0.299
Kidney disease			
Diabetes	3 (18.8)	2 (6.9)	0.226
Hypertensive	0	2 (6.9)	0.283
Glomerular	5 (31.3)	8 (27.6)	0.795
Cystic	3 (18.8)	5 (17.2)	0.899
Other	5 (31.3)	12 (41.4)	0.502
Immunosuppression			
ATG	0	4 (13.8)	0.120
BX	16 (100)	25 (86.2)	0.120
CS	16 (100)	29 (100)	NA
CyA	0	1 (3.4)	0.453
Tac	16 (100)	28 (96.6)	0.453
MMF/MPA	15 (93.8)	29 (100)	0.173
AZA	1 (6.3)	0	0.173
Rejection within first year	3 (18.8)	6 (20.7)	0.876
eGFR at T2 (ml/min/1.73m2)	55.2 (47.2 – 68.6)	43.2 (27.9 – 50.1)	<0.001
Serum-creatinine at T2 (mg/dL)	1.23 (1.07 – 1.54)	1.66 (1.39 – 2.06)	<0.001
Serum-urea at T2 (mg/dL)	49.5 (37 – 54.5)	54 (45 – 79.5)	0.016

Supplementary Table 2. Comparison of intermediate risk CMV+ and CMV- KTRs using Mann-Whitney U test or Chi-Square test, as appropriate. Continuous variables are shown as median and IQR and categorical variables as absolute values and relative frequencies. BMI, Body mass index; HD, hemodialysis; PD, peritoneal dialysis; ECD, extended criteria donor; ATG, anti-thymocyte globulin; BX, basiliximab; CS, corticosteroids; CyA, cyclosporin A; Tac, tacrolimus; MMF, mycophenolate mofetil; MPA, mycophenolic acid; AZA, Azathioprine; eGFR, estimated glomerular filtration rate.

	FoxP3⁺CD25⁺ in CD3⁺CD8⁺ (%)		
	<1.03	>1.03	
N	*20*	*25*	
			**p-Value**
Age (years)	58.5 (45.5 – 64.8)	56 (49.5 – 64.5)	0.855
Caucasian Ethnicity	17 (85)	22 (88)	0.769
Male gender	13 (65)	12 (48)	0.254
BMI (kg/m^2)	26.4 (21.5 – 29.1)	25.2 (21 – 27.4)	0.398
Preemptive	0	3 (12)	0.109
HD/PD	18 (90)/2(10)	18 (72)/4 (16)	0.261/0.556
Diabetes mellitus	4 (20)	2 (8)	0.239
Donor Age (years)	55 (46.3 – 71)	55 (47.5 – 67.5)	0.982
ECD	10 (50)	15 (60)	0.502
BK-Polyoma viremia	4 (20)	4 (16)	0.727
HLA Mismatches			0.330
CMV constellation			0.973
R+/D-	6 (30)	7 (28)	
R+/D+	13 (65)	17 (68)	
R+/D?	1 (5)	1 (4)	
CMV prophylaxis	2 (10)	4 (16)	0.556
Kidney disease			
Diabetes	4 (20)	1 (4)	0.09
Hypertensive	0	2 (8)	0.196
Glomerular	7 (35)	6 (24)	0.419
Cystic	3 (15)	5 (20)	0.663
Other	6 (30)	11 (44)	0.336
Immunosuppression			
ATG	0	4 (16)	0.061
BX	20 (100)	21 (84)	0.061
CS	20 (100)	25 (100)	NA
CyA	0	1 (4)	0.366
Tac	20 (100)	24 (96)	0.366
MMF/MPA	19 (95)	25 (100)	0.258
AZA	1 (5)	0	0.258
Rejection within first year	4 (20)	5 (20)	1
CMV DNAemia	8 (40)	21 (84)	0.002
eGFR at T2 (ml/min/1.73m2)	52.8 (42.6 – 60.9)	45.2 (28.4 – 51.2)	0.064
Serum-creatinine at T2 (mg/dL)	1.51 (1.1 – 1.67)	1.44 (1.28 – 2.06)	0.288

Serum-urea at T2 (mg/dL)	51.5 (41 – 56)	51 (40.5 – 74)	0.552

Supplementary Table 3. Intermediate risk KTRs with frequencies of FoxP3⁺CD25⁺ in CD3⁺CD8⁺ below 1.03% are compared to those with frequencies above 1.03%. Variables are presented as median and IQR or absolute values and relative frequencies, as appropriate. Groups are compared using Chi-square test or Mann-Whitney-U test depending on the tested variable. BMI, Body mass index; HD, hemodialysis; PD, peritoneal dialysis; ECD, extended criteria donor; ATG, anti-thymocyte globulin; BX, basiliximab; CS, corticosteroids; CyA, cyclosporin A; Tac, tacrolimus; MMF, mycophenolate mofetil; MPA, mycophenolic acid; AZA, Azathioprine; eGFR, estimated glomerular filtration rate.
